# Network Analysis and Experimental Verification of the Mechanisms of Hydroxysafflor Yellow A in Ischemic Stroke Following Atherosclerosis

**DOI:** 10.3390/molecules28237829

**Published:** 2023-11-28

**Authors:** Xi Han, Huifen Zhou, Junjun Yin, Jiaqi Zhu, Jiehong Yang, Haitong Wan

**Affiliations:** School of Basic Medical Sciences, Zhejiang Chinese Medical University, Hangzhou 310053, China; m13588897912@163.com (X.H.); zhouhuifen2320@126.com (H.Z.);

**Keywords:** hydroxysafflor yellow A, ischemia stroke following atherosclerosis, network analysis, animal experiments

## Abstract

Hydroxysafflor yellow A (HSYA) is derived from *Carthamus tinctorius* L. (Honghua in Chinese) and is used to treat cardiovascular and cerebrovascular disease. However, the mechanism by which HSYA treats ischemic stroke following atherosclerosis (ISFA) remains unclear. The targets and pathways of HSYA against ISFA were obtained using network analysis. A total of 3335 potential IFSA-related targets were predicted using the GenCards and Drugbank databases, and a total of 88 potential HSYA-related targets were predicted using the Swiss Target Prediction database. A total of 62 HSYA-related targets against IFSA were obtained. The network was composed of HSYA, 62 targets, and 20 pathways. The top 20 targets were constructed via the protein–protein interaction (PPI) network. Gene Ontology analysis revealed that the targets were involved in signal transduction, protein phosphorylation, the cytoplasm, the plasma membrane, the cytosol, zinc ion binding, ATP binding, protein kinase binding/activity, and enzyme binding. The Kyoto Encyclopedia of Genes and Genomes pathway enrichment analysis revealed that the pathways were associated with cancer, inflammatory mediator regulation of the transient receptor potential channels, and microRNA in cancer. Additionally, molecular docking indicated that HSYA mainly interacts with five targets, namely interleukin 1 beta (IL-1β), signal transducer and activator of transcription 3 (STAT3), E1A-binding protein p300 (EP300), protein kinase C alpha (PRKCA), and inhibitor of nuclear factor kappa B kinase subunit beta (IKBKB). In animal experiments, HSYA administration ameliorated the infarct size, neurological deficit score, histopathological changes, carotid intima-media thickness (IMT), and blood lipid level (total cholesterol and triglycerides). Immunochemistry and quantitative PCR showed that HSYA intervention downregulated the expression of STAT3, EP300, PRKCA, and IKBKB, and the enzyme-linked immunoassay showed reduced IL-1β levels. The findings of this study provide a reference for the development of anti-ISFA drugs.

## 1. Introduction

Ischemic stroke (IS), the main type of stroke, is a leading cause of death and disability worldwide [1]. Due to ischemia and hypoxia of brain tissue, IS occurs. Once the vessel is re-opened, the damaged nerve cells undergo secondary damage. This process is called ischemia reperfusion injury [2]. The risk factors for IS include atherosclerosis, hypertension, and diabetes. Atherosclerosis is the primary cause of stroke and an important prognostic factor for recurrent vascular events. Atherosclerosis is fatty-driven and involves the formation of plaques in the artery wall, which leads to thrombosis formation and IS [3]. Thus, atherosclerosis can lead to IS. In the clinic, IS is often accompanied by atherosclerosis. Aspirin has better efficacy in stroke and atherosclerosis [4]; however, aspirin is an antiplatelet agent and can lead to risk of hemorrhage [5].

Traditional Chinese medicine (TCM) has been used to manage human health in China for a long time and has good efficacy in the prevention and treatment of diseases. *Carthamus tinctorius* L. (safflower), a type of TCM, has been widely used for the treatment of cardio-cerebrovascular disease. Hydroxysafflor yellow A (HSYA), the dominant water-soluble component of safflower, exerts pharmacological effects against cerebral ischemia [6] and has anti-oxidative, anti-apoptotic, and anti-inflammatory properties [7,8]. HSYA also reduces the levels of triglycerides (TGs), total cholesterol (TC), and low-density lipoprotein cholesterol, and increases the level of catalase in high-fat diet (HFD)-induced atherosclerosis mice [9]. Therefore, we postulated that HSYA may have efficacy in IS following atherosclerosis (ISFA).

Network analysis is an alternative-systems-level way to identify drug candidates or pharmacodynamic methods [10]. It is constructed using various online public databases to reveal the multilevel relationships between “drug-compound-target-pathway-disease” [11]. Recently, network analysis has been used to elucidate the relationships between the drug–target and disease–target networks [12]. It has become a widely used method to explore or understand the molecular basis of disease, predict the potential mechanisms, and identify the active components of herbs and underlying drug and disease targets.

**Figure 1:**
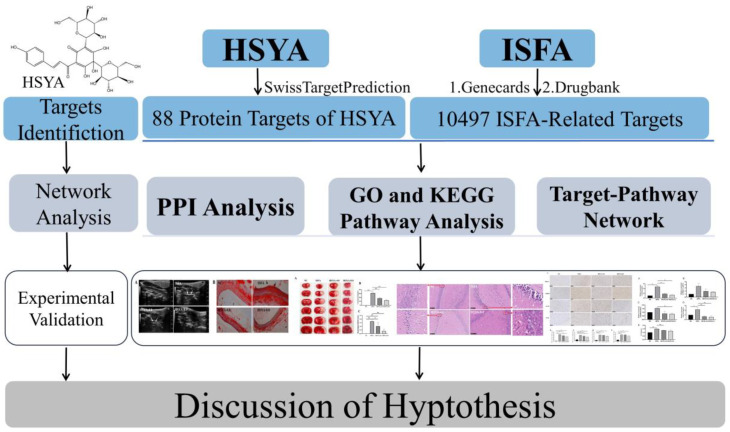
Chemical structure of HSYA and flowchart of the mechanisms of HSYA against ISFA. * *p* < 0.05, ** *p* < 0.01.

In this work, the potential targets, pathways, and molecular docking of HSYA against ISFA were predicted using network pharmacology. To confirm the mechanism of HSYA in ISFA, a rat model of ISFA was established, and various molecular biological methods were used (Figure 1).

## 2. Results

### 2.1. Identification of the Shared Targets between HSYA and ISFA

**Figure 2:**
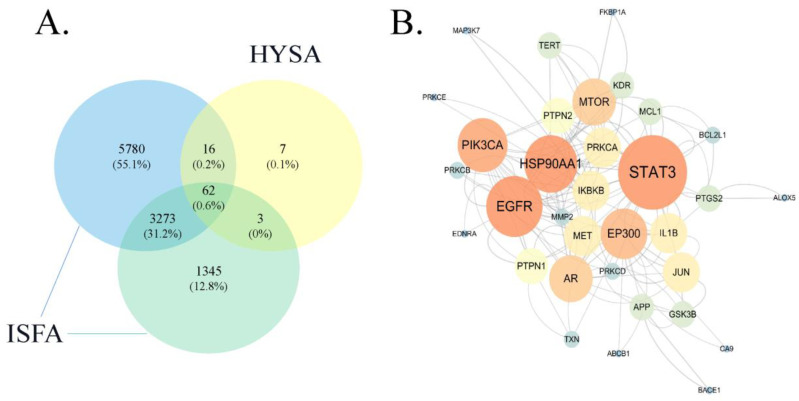
Intersection targets of HSYA and ISFA. (**A**) The blue circle represents the targets of ischemia, the green circle represents targets associated with atherosclerosis, and the yellow circle represents HSYA targets. The overlapping part of the three circles represents shared targets of the drug–disease. (**B**) The PPI network presents protein–protein relationships. The nodes present target proteins.

We screened and identified a total of 4683 atherosclerosis- and 9131 IS-associated targets from the GeneCards and DrugBank databases. A total of 3335 shared targets between atherosclerosis and IS were obtained, and a total of 88 HSYA-related targets were acquired from the SwissTargetPrediction database. Subsequently, 62 drug–disease shared targets were identified using Venn diagrams (Figure 2A). Next, the shared targets were constructed into a PPI network diagram (Figure 2B), and the PPI relationships were analyzed using the STRING database.

### 2.2. HSYA–ISFA Target Pathway Network Construction

**Figure 3:**
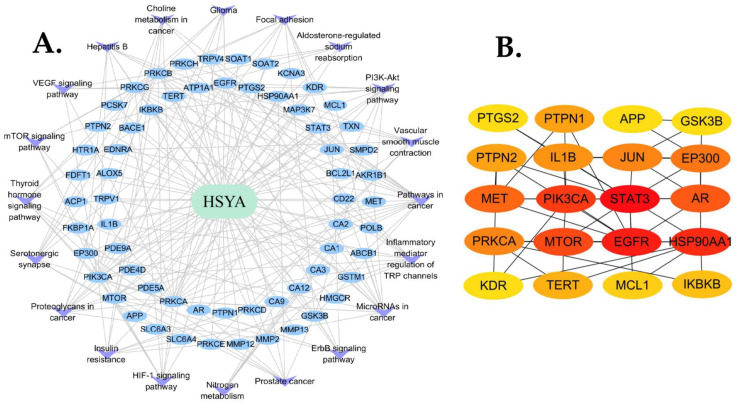
HSYA target pathway network. (**A**) HSYA (aquamarine circles), targets (blue circles), and pathways (purple V shape). (**B**) Network diagram of the top 20 targets of HSYA against ISFA. The darker color refer to the higher ranking.

The HSYA–ISFA target pathway contained a total of 83 nodes. Nodes with different colors and shapes represent active compounds, targets, and pathways, containing HSYA (aquamarine circle), 62 targets (blue circle), and 20 pathways (purple V shape). The number of lines between the nodes indicates the importance of the nodes in the network (Figure 3A).

### 2.3. PPI Network Construction

There were 20 nodes and 37 edges. The Matthews correlation coefficient (MCC) score is shown in Table 1. As shown in Figure 3B, the top 20 targets of HSYA against ISFA were as follows: heat shock protein 90 alpha family class A member 1, IL-1β, E1A-binding protein p300 (EP300), protein kinase C alpha (PRKCA), IkappaB kinase beta (IKBKB), signal transducer and activator of transcription 3 (STAT3), phosphatidylinositol-4,5-bisphosphate 3-kinase catalytic subunit alpha, epidermal growth factor receptor, mechanistic target of rapamycin kinase, androgen receptor, MET proto-oncogene receptor tyrosine kinase, Jun proto-oncogene, AP-1 transcription factor subunit, protein tyrosine phosphatase non-receptor type 2 (PTPN2), PTPN1, telomerase reverse transcriptase, MCL1 apoptosis regulator, amyloid-beta precursor protein, glycogen synthase kinase 3 beta, kinase insert domain receptor, and prostaglandin-endoperoxide synthase 2.

### 2.4. GO and KEGG Pathway Enrichment Analyses

**Figure 4:**
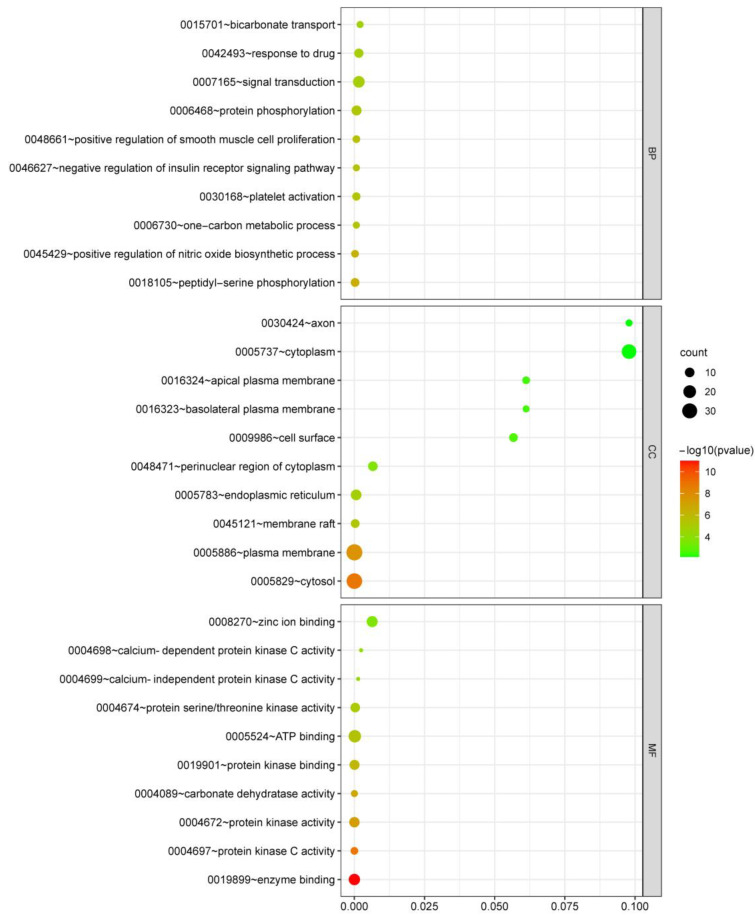
Bubble chart results of GO enrichment analysis of BP, CC, and MF.

**Figure 5:**
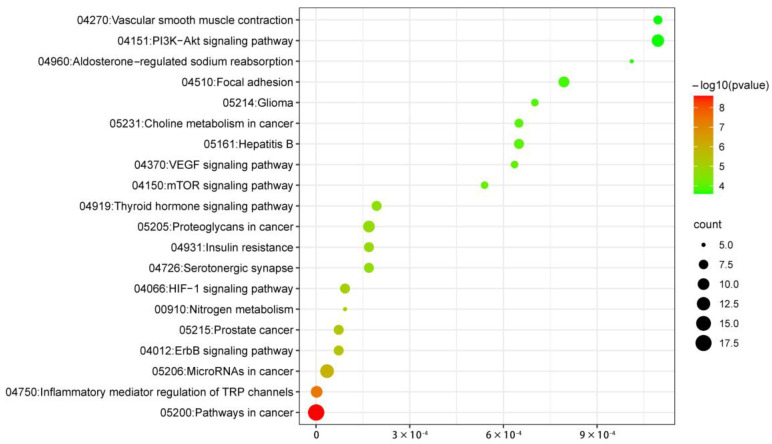
Bubble chart results of the top 20 signaling pathways from the KEGG enrichment analysis.

To gain detailed insights into the biological features of potential targets of HSYA in ISFA, GO (Gene Ontology) enrichment and Kyoto Encyclopedia of Genes and Genomes (KEGG) pathway analyses were conducted using the bioinformatics platform. As shown in Figure 4, the top 10 GO terms were significantly selected in the BP, CC, and MF categories, indicating that HSYA may regulate the IFSA process mainly via signal transduction, protein phosphorylation, the cytoplasm, the plasma membrane, the cytosol, zinc ion binding, ATP binding, protein kinase binding/activity, and enzyme binding. In addition, to investigate the underlying pathways of HSYA against ISFA, KEGG pathway analysis showed that the top 20 pathways were mainly in cancer, inflammatory mediator regulation of the transient receptor potential (TRP) channels, and microRNA in cancer (Figure 5).

### 2.5. Molecular Docking 

**Figure 6:**
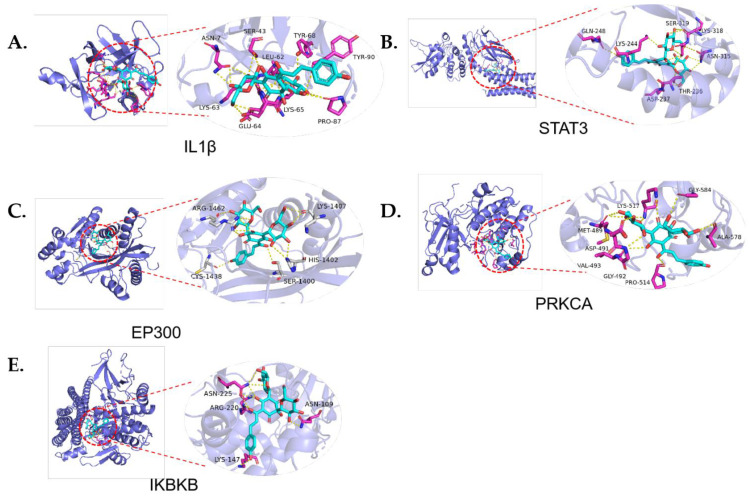
Results of molecular docking between HSYA and targets are shown. (**A**) IL-1β, (**B**) STAT3, (**C**) EP300, (**D**) PRKCA, (**E**) IKBKB. The blue sticks indicate HYSA.

HSYA mainly interacted with the binding sites of ASN-7, SER-43, LEU-62, TYR-68, TYR-90, LYS-63, GLU-64, LYS-65, and PRO-87 in IL-1β; of ARG-1462, LYS-1407, CYS-1438, SER-1400, and HIS-1402 in EP300; of ASN-225, ASN-109, ARG-220, and LYS-147 in IKBKB; of GLN-248, LYS-244, SER-319, LYS-318, ASN-315, THR-236, and ASP-237 in STAT3; and of VAL-493, ASP-491, MET-489, LYS-517, GLY-584, ALA-578, PRO-514, and GLY-492 in PRKCA. Our molecular docking results revealed the efficient binding of HSYA with targets, namely IL-1β, EP300, PRKCA, IKBKB, and STAT3 (Figure 6).

### 2.6. Assessment of Carotid Ultrasound and Effects of HSYA on Arterial Plaque

**Figure 7:**
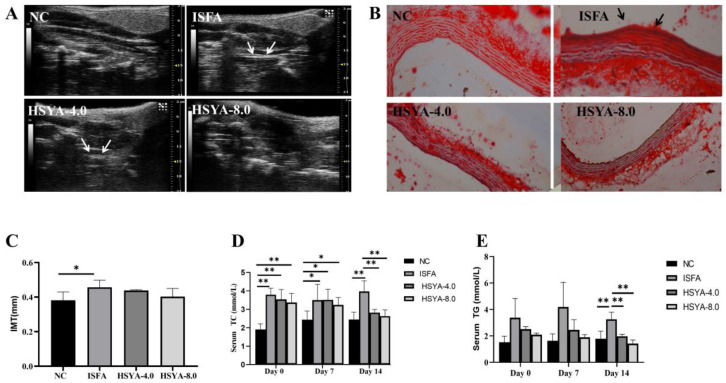
HSYA administration improved the carotid IMT and blood lipid levels in rats. The effects of HSYA on blood lipid levels on days 0, 7, and 14 were evaluated. (**A**) Representative carotid ultrasound images of IMT were measured in each rat. The white arrows represent an echo plaque. (**B**) Arterial Oil Red O staining. The black arrow shows that the ISFA group exhibits more pronounced neck plaques compared to the other groups. (**C**) The values of IMT were quantified. (**D**,**E**) Serum levels of TC and TG were measured. Data are expressed as $\bar{x}$ ± SEM, n = 3 per group. * *p* < 0.05, ** *p* < 0.01.

To investigate whether the rat models of atherosclerosis were successfully established, all rats underwent carotid ultrasound at 8 weeks. After 14 days of administrations, the rats underwent carotid ultrasound again at 10 weeks. The ultrasound results demonstrated that compared with the NC group, the carotid IMT was significantly increased in the ISFA group (*p* < 0.05; Figure 7A,C). Compared with the ISFA group, the carotid IMT was decreased in the HSYA 8.0 treatment group. As shown in Figure 7B, there were some plaque in the model group, and no plaques in the NC and two HSYA groups. 

### 2.7. Effects of HSYA on Blood Lipid Levels

Figure 7D shows that on day 0 after middle cerebral artery occlusion (MCAO), the levels of TC in the NC group were significantly lower than those in the ISFA group and the two HSYA groups (*p* < 0.05). On day 14, the levels of TC in the NC group were significantly lower than those in the ISFA group (*p* < 0.01), and the TC levels in the two HSYA groups were significantly lower than those in the ISFA group (*p* < 0.01). 

Figure 7E shows that on day 7, the levels of TG in the NC group were lower than those in the ISFA group. Additionally, the levels of TC and TG in the two HSYA groups were lower than those in the ISFA group. Until day 14, the levels of TG in the NC group were significantly lower than those in the ISFA group (*p* < 0.01), and the levels of TG in the two HSYA groups were significantly lower than those in the ISFA group (*p* < 0.01).

### 2.8. Assessment of Rat Models of MCAO

**Figure 8:**
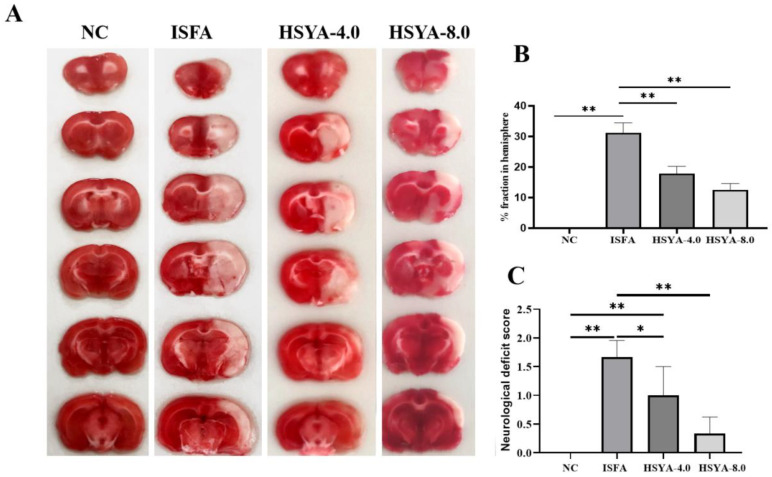
HSYA administration reduced the cerebral infarct size and neurological deficit score in rats with ISFA. (**A**) Representative images of TTC staining in different groups. Rat brains were stained using TTC. (**B**) Quantitation of TTC staining of brain sections (infarct size of each group). (**C**) The evaluation of neurological deficit. Data are expressed as $\bar{x}$ ± SEM, n = 3 per group. * *p* < 0.05, ** *p* < 0.01.

To assess the rat models of MCAO, 2,3,5-triphenyltetrazolium chlorides (TTC) staining was conducted. The infarct size of the NC group (deep red) was significantly smaller than that of the ISFA group (*p* < 0.01; Figure 8A,B). Compared with the ISFA group, the infarct size of the HSYA group (4 or 8 mg/kg/d) was reduced (*p* < 0.01). To evaluate the effects on the treatment of ISFA, a neurological deficit score was used. As shown in Figure 8C, the scores of the ISFA group were significantly higher than those of the NC group (*p* < 0.01), and, compared with the ISFA group, the scores of the HSYA group were significantly decreased (*p* < 0.01 or *p* < 0.05). 

### 2.9. Effects of HSYA on the Histopathological Changes in Rats with ISFA

**Figure 9:**
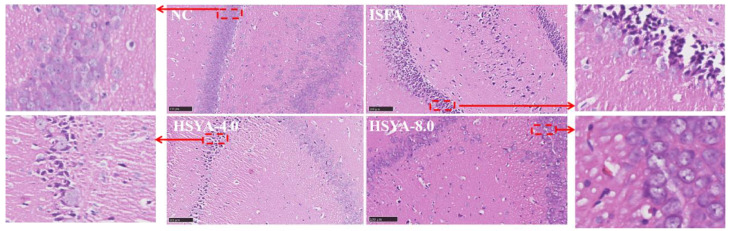
Effect of HSYA on the pathological changes in brain tissue. Representative images of HE staining (bar = 100 μm) are shown.

As shown in Figure 9, there were no histopathological abnormalities in the NC group, and the tissue structures were neatly arranged, with obvious nucleoli and without intracellular edema. The ISFA group had swollen cells and a loosely arranged structure. Additionally, edematous cells, a loose cytoplasm, and severe cell deformation were observed in the ISFA group. HSYA administration reduced these histopathological changes.

### 2.10. Effects of HSYA on the Expression of PRKCA, IKBKB, STAT3, and NF-κB in Rat Brain Tissue

**Figure 10:**
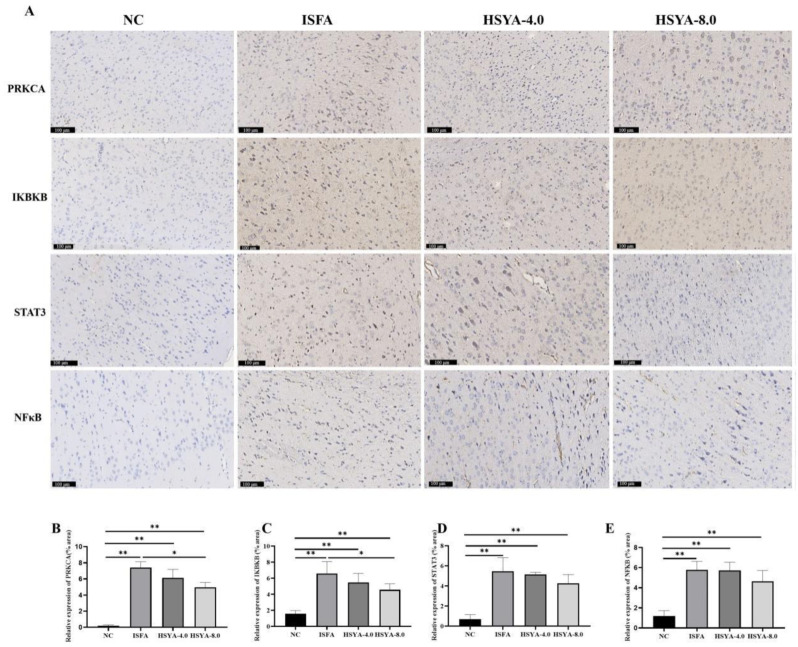
HSYA administration decreased the expressions of PRKCA, IKBKB, STAT3, and NF-κB in rats with IFSA. (**A**) Representative images of staining of PRKCA, IKBKB, STAT3, and NF-κB (bar = 100 μm). (**B**–**E**) Quantitative analysis for immunohistochemical staining of PRKCA, IKBKB, STAT3, and NF-κB. Data are expressed as $\bar{x}$ ± SEM, n = 3 per group. * *p* < 0.05, ** *p* < 0.01.

To assess the expression of PRKCA, IKBKB, STAT3, and NF-κB, immunochemical staining was conducted. As shown in Figure 10, compared with the NC group, the expression of PRKCA, IKBKB, STAT3, and NF-κB was significantly increased in the ISFA group (*p* < 0.01), and the expression of PRKCA and IKBKB in the HSYA 8.0 treatment group was significantly lower than that in the ISFA group (*p* < 0.05).

### 2.11. Effects of HSYA on the mRNA Levels of EP300, PRKCA, IKBKB, and STAT3 in Rat Brain Tissue

**Figure 11:**
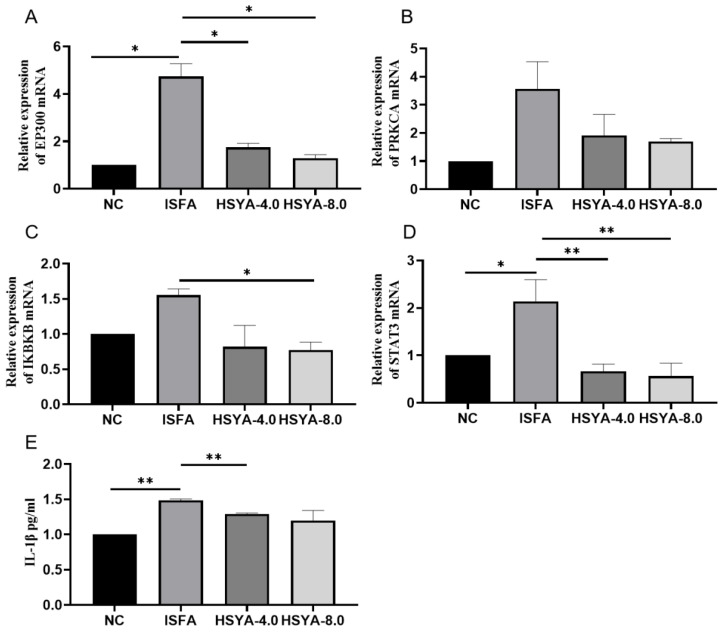
HSYA administration reduced mRNA levels of EP300, PRKCA, IKBKB, and STAT3 in brain tissue and IL-1β levels in serum. (**A**) EP300, (**B**) PRKCA, (**C**) IKBKB, (**D**) STAT3, and (**E**) IL-1β. Data are expressed as $\bar{x}$ ± SEM, n = 3 per group. * *p* < 0.05; ** *p* < 0.01.

To determine the mRNA levels of EP300, PRKCA, IKBKB, and STAT3, quantitative PCR (qPCR) was carried out. Compared with the NC group, the mRNA levels of EP300, PRKCA, IKBKB, and STAT3 were significantly increased (*p* < 0.01; Figure 11A–D) in the ISFA group. There were significant decreases in EP300 and STAT3 in the two treatment groups and IKBKB in the HSYA 8.0 treatment group, compared with the ISFA group (*p* < 0.01 or *p* < 0.05).

### 2.12. Effects of HSYA on the IL-1β Level in Rat Serum

Compared with the NC group, the IL-1β level was increased in the ISFA group, whereas there was a significant decrease in the HSYA 4.0 treatment group compared with the ISFA group (*p* < 0.01; Figure 11E).

## 3. Discussion

In recent years, network analysis has been used to investigate the relationship between drugs and diseases. The main results of this study were as follows. (1) Based on network analysis, HSYA was used to treat ISFA. The results showed that HSYA could regulate the 62 targets associated with ISFA, including PRKCA, IKBKB, STAT3, EP300, IL-1β, and NF-κB. These 62 targets were mainly enriched in cancer, inflammatory mediator regulation of the TRP channels, and microRNA in cancer. In addition, the AutoDock Vina 1.2.0 docking program was used to simulate the binding position between HSYA and the key genes. The docking results directly show the binding site and method and the type and number of amino acid residues. The binding energies between the targets and HSYA were less than −7.7 kcal/mol (Table 2), so 5 of the top 20 targets were selected (Figure 6). However, HSYA is a small molecule that tends to exhibit a non-specific “docking” effect, and more experiments are needed to confirm the results. (2) The animal experiments demonstrated that HSYA can improve the infarct volume of ISFA, carotid IMT, and blood lipid levels. Oil Red O staining of the artery was reduced by HSYA administration. The immunohistochemical results showed that HSYA regulated the positive expression of PRKCA, IKBKB, STAT3, and NF-κB. Additionally, the qPCR results revealed a significant increase in the mRNA expression of EP300, PRKCA, IKBKB, and STAT3 in the ISFA group compared with the NC group, and decreased expression in the two treatment groups compared to the ISFA group. The ELISA results showed that HSYA administration reduced the IL-1β levels.

**Figure 12:**
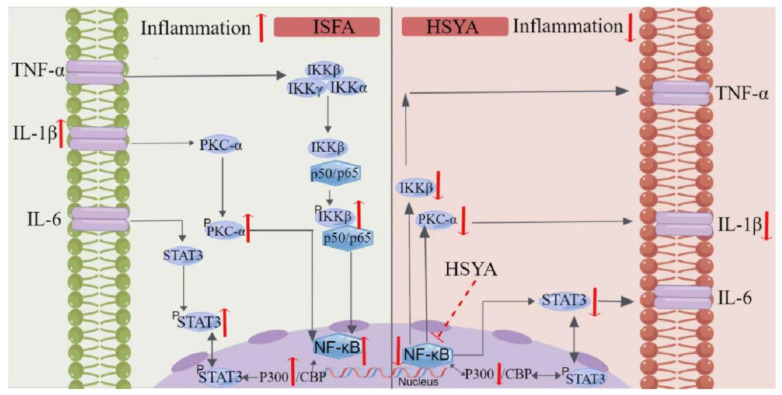
Schematic illustration of the mechanism of action of HSYA in ISFA (Arrow up represented up-regulation, and arrow down represented down-regulation).

PRKCA, known as PKC-α, is a member of the PKC family of serine/threonine kinases [13]. Hang Yu et al. [14] reported that angiopoietin-1 intervention reduces the loss function of the blood–brain barrier in rats with cerebral ischemia, and also downregulates PKC-α expression, indicating that reduced PKC-α expression contributes to improving cerebral ischemic events. The expression of PKC-α in the NC group was lower than that in the ISFA group, and the PKC-α expression of the two treatment groups was decreased compared with the ISFA group, consistent with prior work. The knockdown of PKC-α enhances tumor necrosis factor-alpha expression and the inhibition of PKC-α inactivates NF-κB [15]. The IκB kinase complex controls NF-κB gene transcription as a signaling hub [16]. The deficiency in IKKβ helps reduce neuronal cell death, downregulates IL-1β expression, and increases microglial recruitment [17,18]. Neuronal IKKβ/NF-κB activation inhibits insulin and leptin signaling, enhancing food intake and body weight gain in HFD-fed mice [19]. We found that the IKKβ, IL-1β, and NF-κB expression in the HSYA group was significantly lower than that in the IFSA group, consistent with previous studies. STAT3 is a transcription factor that exerts vital functions in NF-κB inflammatory pathways [20]. STAT3 is phosphorylated by p-JAK2 and then translocates to the nucleus. JAK2/STAT3 pathway activation improves IS events [21], which may be associated with angiogenesis [22]. The JAK2/STAT3 pathway leads to angiogenesis resulting from vascular endothelial growth factor (VEGF) production [23]. Furthermore, JAK2/STAT3 pathway activation changes the microglia to M_2_ polarization to promote angiogenesis in IS, and hypoxia-inducible factor 1 alpha and VEGF expression is regulated [24]. In our study, the results showed that the STAT3 expression in the HSYA group was significantly lower than that in the IFSA group, indicating that HSYA administration improves ISFA. The molecular mechanisms of these key targets are shown in Figure 12 and are consistent with the findings of several independent studies. This study focused on a preliminary exploration of the efficacy of HSYA in ischemic stroke following atherosclerosis (ISFA) models. However, further research is required to investigate the underlying mechanism in more detail.

## 4. Materials and Methods

### 4.1. Identifying HSYA Genes Associated with ISFA

All putative HSYA genes were collected from online databases [25].At the same time, ISFA-associated pharmacological genes were obtained from the GeneCards and Drugbank databases. Genes associated with HSYA and ISFA were subjected to intersection analysis using Venn diagrams (http://bioinformatics.psb.ugent.be/webtools/Venn/ (accessed on 1 September 2022)) to identify their shared genes.

### 4.2. Construction of the Protein–Protein Interaction Network 

The selected genes between HSYA and ISFA were input into the STRING (https://string-db.org/ (accessed on 22 January 2022)) database [26] for protein–protein interaction (PPI) network analysis. Proteins at a minimum required interactions with a confidence score ≥ 0.9. The Cytoscape 3.7.1 software was used to visualize the PPI network. Based on the cytoHubba plugin, the top 20 hub genes were identified, and potential genes were predicted.

### 4.3. Gene Ontology and Kyoto Encyclopedia of Genes and Genomes Pathway Enrichment Analyses

The potential hub genes of HSYA against ISFA were submitted into DAVID Bioinformatics Resources 6.8 [27], and Gene Ontology (GO) and Kyoto Encyclopedia of Genes and Genomes (KEGG) pathway enrichment analyses [28] were conducted. GO enrichment analysis generally includes three ontologies: biological process (BP), cellular component (CC), and molecular function (MF). The statistical significance threshold was set at *p* < 0.01. A corresponding bubble diagram was plotted using the bioinformatics platform (http://www.bioinformatics.com.cn (accessed on 30 July 2022)), a free online platform for data analysis and visualization.

### 4.4. Chemicals and Reagents

#### 4.4.1. Preparing for Animals

Healthy male Sprague-Dawley (SD) rats, weighing 400–430 g, were purchased from Laboratory Animal Research Center, Zhejiang Chinese Medicine University (quality certificate no. SYXK 2021-0012). All rats were housed in an SPF-rated facility with 12/12 h dark/light. All rats were given free access to standard feed and purified water and retained for 7 days. All procedures were carried out in accordance with the regulations of the Animal Ethics Committee of Zhejiang Chinese Medicine University (Animal Ethics Application number: 20210103-0130. Approval number: IACUC-20210406-06). All animal experiments in the study were approved by the Institutional Animal Care and Use Committee at Zhejiang Chinese Medical University and complied with the Animal Management Guidance of the Chinese Ministry of Health and existing current animal welfare guidelines.

#### 4.4.2. Construction of a Rat Model of ISFA 

The rats were divided into two groups and fed for 7 days: a normal control (NC) group (basic diet) and a model group (HFD and intraperitoneal injection of 600,000 IU/kg vitamin D3 after 2 days). The HFD was composed of the following: 49% standard diet, 17% lard, 3% sesame oil, 9% sucrose, 8% whole milk powder, 8% casein, 2% premix, 2% calcium hydrogen phosphate, 1.5% cholesterol, and 0.5% sodium cholate. After two months, the blood samples were collected from the submaxillary vein to measure the serum levels of TC and TG. Carotid ultrasonography was used to monitor the intima-media thickness (IMT). Oil Red O staining was used to assess aortic plaque. Compared with the NC group, the plasma lipid levels and IMT in the HFD group were significantly different (*p* < 0.05), and aortic plaque was present, indicating that an atherosclerosis rat model was successfully established. Subsequently, middle cerebral artery occlusion (MCAO) was carried out. The rats were randomly assigned to four groups (n = 9 per group): NC, ISFA, HSYA 4.0 (4 mg/kg/d), and HSYA 8.0 (8 mg/kg/d). The NC group and ISFA group were given the same amount of saline for injection. The drug administration method was slow intravenous administration via the caudal vein for 14 consecutive days. There were 36 rats used in this study. 

### 4.5. Neurological Scores

At 24 h after MCAO, neurological scores were evaluated according to the Zea Longa [29] 5-point scoring method: 0, no signs of nerve injury; 1, loss of the ability to fully stretch the contralateral forepaw; 2, the animal turns to one side while walking; 3, disabled crawls; and 4, a loss of consciousness. In this study, rats that achieved scores between 1 and 3 were considered successful models.

### 4.6. Carotid Ultrasound and Imaging [30]

All rats underwent carotid ultrasound examination using the Vevo 1100 Small Animal Ultrasonic Sound Imaging System with a 15 MHz linear array transducer (VisualSonics, Toronto, ON, Canada). An optimal two-dimensional image of the common carotid artery was obtained, and the IMT was measured. All measurements were repeated at least three times.

### 4.7. 2,3,5-Triphenyltetrazolium Chloride Staining (TTC)

The rats were euthanized at 24 h following MCAO. The cerebral tissues were collected and frozen at −20 °C for 15 min and then sliced into 2 mm thick coronal sections. The sections were stained using 2% TTC-PBS solution for 15 min at 37 °C to differentiate ischemic from non-ischemic regions. The staining results were photographed and analyzed using the ImageJ software (version 1.52). The infarct volume was calculated as follows: infarct volume (%) = (infarct volume of sections)/(total volume of sections) × 100%.

### 4.8. Biochemical Detection in Serum 

The TC level was measured using a cholesterol total kit (CHOD-PAP substrate method, Shanghai, China) in a 3100 automatic analyzer (Hitachi, Tokyo, Japan) and the TG level was measured using a triglyceride kit (GPO-PAP method, Qingdao, China) in a 3100 automatic analyzer.

### 4.9. Quantitative Real-Time PCR (RT-qPCR)

The total RNA was extracted from the ischemic area of the brain tissues using the Tissue Total RNA Isolation Kit (Vazyme FastPure^®^ Cell/Tissue Total RNA Isolation Kit, V2, RC112). The RNA was reverse-transcribed into cDNA using a 5× All-In-One RT MasterMix kit. The procedure was as follows: incubate the tube at 25 °C for 10 min, 42 °C for 15 min, and 85 °C for 5 min. Subsequently, quantitative real-time PCR (RT-qPCR) was performed using a 2× Universal SYBR Green Fast qPCR Mix kit (2× Universal SYBR Green Fast qPCR Mix). The program steps were as follows: denaturation at 95 °C for 3 min (one cycle), 42 cycles at 95 °C for 5 s and 60 °C for 30 s. The melting curve was automatically set. The relative expression was normalized to glyceraldehyde 3-phosphate dehydrogenase (GAPDH). All values were calculated using the 2^−ΔΔCt^ method. The premiers of EP300, PRKCA, STAT3, and IKBKB were listed in Table 3. 

### 4.10. Enzyme-Linked Immunosorbent Assay 

The serum levels of interleukin 1 beta (IL-1β) were measured using an enzyme-linked immunoassay (ELISA) kit (Jiangsu Mei Biao Biological Technology Co., Ltd., Nantong, China) according to the manufacturer’s instructions. Briefly, the procedure was as follows: the detection antibody with specimens, standard samples, and HRP marks was added to a pre-coated micro-well plate. The optical density (OD) was measured at a wavelength of 450 nm.

### 4.11. Hematoxylin and Eosin, Immunohistochemical Staining, and Oil Red O Staining

Briefly, the rat brain tissue samples of all groups were soaked in 4% paraformaldehyde for 72 h. After dehydration, washing, infiltration, and embedding, the tissues were cut into 4 μm thick sections and stained using hematoxylin and eosin to observe the pathological changes.

Immunohistochemistry (IHC) staining was performed to measure the expressions in the brain tissues of the rats. The sections were incubated with PRKCA (1:100, Proteintech, San Diego, CA, USA, 21991-1-AP), STAT3 (1:100, Proteintech, USA, 10253-2-AP), IKBKB (1:100, Proteintech, USA, 15649-1-AP), and NF-κB (1:100, Proteintech, USA, 10745-1-AP) overnight at 4 °C, followed by incubation with an HRP-marked goat anti-rabbit antibody (1:500, Dawn Biotec, Brisbane, CA, USA, DW-GAR007) for 2 h at room temperature. Finally, the sections were observed under an optical microscope (NanoZoomer 2.0 RS, Hamamatsu Photonics, Hamamatsu, Japan).

For Oil Red O Staining, the aorta was fixed using 4% paraformaldehyde for 24 h, dehydrated with 30% sucrose for 1 h, embedded with OCT, and frozen in slices. Then, the slices were stained using Oil Red O for 10 min, and hematoxylin counterstained for 3 min. The slices were observed under an optical microscope.

### 4.12. Statistical Analysis

All data were analyzed using the SPSS 25.0 software, and are expressed as the mean ± SEM. One-way analysis of variance followed by Tukey’s test for comparisons between more than two groups was used for the statistical analysis. The statistical differences between two groups were analyzed using Student’s *t*-test. Differences were considered statistically significant at *p* < 0.05.

## 5. Conclusions

The present study revealed that HSYA has protective effects against ISFA. We obtained potential targets associated with ISFA using network analysis. Animal models of MCAO combined with atherosclerosis showed that the neurological function and the volume of cerebral infarction of rats treated with HSYA were improved. Meanwhile, the carotid IMT was also reduced by the administration of HSYA. HSYA administration downregulated the expression of IL-1β, EP300, PRKCA, IKBKB, and STAT3. This study provides novel insights for the development of anti-ISFA drugs.

## Figures and Tables

**Table 1:** Top 20 in network STRING interactions (ranked by MCC method).

Rank	Name	Score	Rank	Name	Score
1	STAT3	60	11	IL1B	10
2	EGFR	50	12	PTPN2	7
3	HSP90AA1	31	12	PTPN1	7
4	PIK3CA	29	14	TERT	6
5	MTOR	25	15	IKBKB	5
6	AR	20	16	MCL1	4
7	MET	18	17	PTGS2	3
8	EP300	16	17	APP	3
9	JUN	14	17	KDR	3
9	PRKCA	14	17	GSK3B	3

**Table 2:** Binding energy for core targets.

Target	Binding Energy (kcal/mol)	Target	Binding Energy (kcal/mol)
IL-1β	−7.7	STAT3	−8.1
EP300	−8.1	PRKCA	−8.4
IKBKB	−8.1		

**Table 3:** The premiers of EP300, PRKCA, STAT3, and IKBKB.

Target	Forward (5′ to 3′)	Reverse (5′ to 3′)
EP300	ACCTTCTCCTGTTCCTAGCCGTAC	AATTGCTGTTGCTGCTGGTTGTTG
PRKCA	TCCCTTTCCTTCGGCGTCTCAG	CGTTGCCTTCTTCATCTCCTTCTGG
STAT3	AGGGCTTCTCGTTCTGGGTCTG	CTCCCGCTCCTTGCTGATGAAAC
IKBKB	CTGGTAGAACGGATGATGGCACTG	TGGCTTCTCCCTGAGTCTTCTGTAG

## Data Availability

The data presented in this study are available on request from the corresponding author. The data are not publicly available due to privacy considerations.

## Abbreviations

Ischemic stroke, IS; hydroxysafflor yellow A, HSYA; traditional Chinese medicine, TCM; middle cerebral artery occlusion, MCAO; triglycerides, TG; total cholesterol, TC; high-fat diet, HFD; ischemic stroke following atherosclerosis, ISFA; Gene Ontology, GO; Kyoto Encyclopedia of Genes and Genomes, KEGG; protein–protein interaction, PPI; 2,3,5-triphenyltetrazolium chlorides, TTC; E1A-binding protein p300, EP300); protein kinase C alpha, PRKCA; IkappaB kinase beta, IKBKB; intima-media thickness, IMT; Matthew’s correlation coefficient, MCC.
